# Clinical Features vs CT Findings to Estimate Need for Surgery in Small Bowel Obstruction

**DOI:** 10.1001/jamanetworkopen.2023.41376

**Published:** 2023-11-02

**Authors:** Sara Schulwolf, Charles Brower, Alessandra Karam, Joaquim Havens, Hamid Shokoohi, Nicole M. Duggan, Andrew J. Goldsmith

**Affiliations:** 1 University of Connecticut School of Medicine, Farmington; 2 University of Cincinnati Emergency Medicine Residency, University of Cincinnati College of Medicine, Cincinnati, Ohio; 3 Central Michigan University College of Medicine, Mt Pleasant; 4 Department of Surgery, Brigham and Women’s Hospital, Harvard Medical School, Boston, Massachusetts; 5 Department of Emergency Medicine, Massachusetts General Hospital, Harvard Medical School, Boston; 6 Department of Emergency Medicine, Brigham and Women’s Hospital, Harvard Medical School, Boston, Massachusetts

## Abstract

This comparative effectiveness research compares the ability of clinical features vs clinical features with computed tomography (CT) to estimate the need for small bowel obstruction surgery.

## Introduction

Approximately 20% to 30% of patients hospitalized with small bowel obstruction (SBO) fail nonoperative treatment.^1,2^ Rapidly identifying patients requiring urgent surgical intervention is critical.^3^ Currently, computed tomography (CT) is the criterion standard for diagnosing SBO management. However, practice variation exists due to a lack of definitive operative radiographic findings for SBO.^4^ We compared the ability of clinical features alone with that of clinical features combined with CT findings to estimate the need for surgical SBO management.

## Methods

Approval for this comparative effectiveness research, including a waiver for informed consent because participants were subject to no more than minimal risk, was granted by the Mass General Brigham institutional review board. This study is reported following the ISPOR reporting guideline.

To compare the ability of clinical features alone with that of CT findings in combination with clinical features to estimate the need for SBO surgery, we performed a retrospective multicenter electronic health record review of adults (aged ≥18 years) presenting to 10 academic and community emergency departments from 2017 to 2020. Patients with a diagnosis code for SBO and with CT-confirmed SBO were included. There were 22 clinical characteristics and 16 CT findings used in this statistical analysis (Table). The decision for surgical management was made by the clinical attending surgeon. We applied χ^2^ and Fisher exact testing and Wilcoxon rank sum to examine associations between categorical and continuous variables, respectively. Least absolute shrinkage and selection operator (LASSO) was used to compare the value of clinical characteristics alone vs clinical characteristics plus CT findings to estimate the need for operative intervention. All statistical tests were performed in SAS statistical software version 9.4 (SAS Institute) and were considered significant at the 95% confidence limit. Data were analyzed from March 2021 to February 2023.

**Table.** Significant Clinical and Imaging Characteristics^a^

Characteristic	Patient encounters, No. (%) (N = 4478)		
	Surgery within 24 h (n = 463 [10.3%])	Surgery within 48 h (n = 575 [12.8%])	Surgery during admission (n = 962 [21.5%])
Age, median (IQR), y	66 (53-76)	67 (55-77)	67 (55-76)
BMI, median (IQR)	25.8 (22.3-30.9)	25.7 (22.2-30.7)	25.2 (21.6-29.6)
Clinical comorbidity index, median (IQR)	1.0 (<0.1-2.0)	1.0 (<0.1-2.0)	1.0 (<0.1-3.0)
Sex			
Female	259 (55.0)	321 (55.8)	546 (56.8)
Male	204 (44.1)	254 (44.2)	416 (43.2)
Medical history			
Abdominal or pelvic surgery	354 (76.5)	441 (76.7)	764 (79.4)
Malignant neoplasm	137 (29.6)	178 (31.0)	370 (38.5)
Genitourinary	23 (5.0)	30 (5.2)	60 (6.2)
Gynecological	28 (6.0)	32 (5.6)	85 (8.8)
GI	45 (9.7)	64 (11.1)	153 (15.9)
SBO	112 (24.2)	153 (26.6)	314 (32.6)
Inflammatory bowel disease	29 (6.3)	36 (6.3)	62 (6.4)
Patient-reported history of present illness			
Bloating or abdominal distension	112 (24.2)	151 (26.3)	273 (28.4)
Obstipation	128 (27.6)	158 (27.5)	277 (28.8)
Physical exam			
Abdominal guarding	97 (21.0)	103 (17.9)	124 (12.9)
Rebound tenderness	40 (8.6)	41 (7.1)	51 (5.3)
Abdominal tenderness	414 (89.4)	499 (86.8)	809 (34.3)
Tap or shake tenderness	61 (13.2)	66 (11.5)	78 (8.1)
Hernia present	105 (22.7)	132 (23.0)	181 (18.8)
Initial vital signs, median (IQR)			
Heart rate, beats/min	84 (75-99)	84 (72-100)	86 (74-100)
Systolic blood pressure, mm Hg	139 (122-161)	138 (122-160)	136 (121-158)
Respiratory rate, breaths/min	18 (17-20)	18 (18-20)	18 (18-20)
Room air oxygen saturation, %	98.0 (96.0-99.0)	98.0 (96.0-99.0)	96.5 (96.0-99.0)
Laboratory values from initial lab draw, median (IQR)			
Sodium, mEq/L	139 (136-141)	139 (136-141)	139 (136-141)
Bicarbonate, mEq/L	25 (22-27)	25 (22-27)	25 (23-27)
Hemoglobin, g/dL	13.8 (12.4-15.2)	13.8 (12.3-15.2)	13.6 (12.0-15.1)
Lactic acid maximum value, mmol/L	1.6 (1.1-2.4)	1.5 (1.1-2.3)	1.5 (1.1-2.1)
CT imaging characteristics			
Partial SBO	40 (8.6)	55 (9.6)	106 (11.0)
High-grade obstruction	90 (19.4)	115 (20.0)	180 (18.7)
Closed-loop obstruction	155 (33.5)	166 (28.9)	194 (20.2)
Mesenteric swirl	83 (17.9)	92 (16.0)	108 (11.2)
Dilated bowel loops	29 (6.3)	34 (5.9)	49 (5.1)
Bowel wall hypoenhancement	40 (8.6)	40 (7.0)	47 (4.9)
Pneumatosis intestinalis	13 (2.8)	14 (2.4)	15 (1.6)
Small bowel fecalization	81 (17.5)	99 (17.2)	162 (16.8)
Mesenteric hyperemia	65 (14.0)	76 (13.2)	99 (10.3)
Mesenteric stranding or inflammation	163 (35.2)	183 (31.8)	267 (27.8)
Interloop fluid	74 (16.0)	84 (14.6)	127 (13.2)
Free fluid	257 (55.5)	310 (53.9)	519 (54.0)
Free air	20 (4.3)	20 (3.5)	28 (2.9)
Hernia	121 (26.1)	154 (26.8)	208 (21.6)
Internal hernia	92 (19.9)	97 (16.9)	115 (12.0)
Obstructing tumor or peritoneal carcinomatosis	23 (5.0)	40 (7.0)	136 (14.1)

Abbreviations: BMI, body mass index (calculated as weight in kilograms divided by height in meters squared); CT, computed tomography; GI, gastrointestinal; SBO, small bowel obstruction.

SI conversion factors: To convert bicarbonate and sodium to millimoles per liter, multiply by 1.0; hemoglobin to grams per liter, multiply by 10.0.

^a^ Significance was determined by (least absolute shrinkage and selection operator) LASSO.

## Results

Of 4478 cases of confirmed SBO, surgical management was required within 24 hours of presentation in 463 cases (10.3%), 48 hours of presentation in 575 cases (12.8%), and at any time during the patient’s index hospital admission in 962 cases (21.5%). A model combining 22 clinical features alone achieved the same performance (area under the receiver operating characteristic curve [AUROC]) in estimating the need for surgical SBO management as a model combining the same clinical features with 16 CT findings (Table) within 24 hours (AUROC [SD], 0.79 [0.01]; 95% CI, 0.77-0.82 vs AUROC [SD], 0.78 [0.01]; 95% CI, 0.76-0.81; *P* = .72) (Figure, A) and 48 hours (AUROC [SD], 0.77 [0.01]; 95% CI, 0.75-0.80 vs AUROC [SD], 0.77 [0.01]; 95% CI, 0.75-0.80; *P* = .82) (Figure, B) of presentation. Clinical features alone vs clinical features with CT findings were more accurate in estimating the need for surgery at all times (AUROC [SD], 0.75 [0.01]; 95% CI, 0.73-0.78 vs AUROC [SD], 0.54 [0.02]; 95% CI, 0.51-0.57; *P* < .001).

**Figure.**
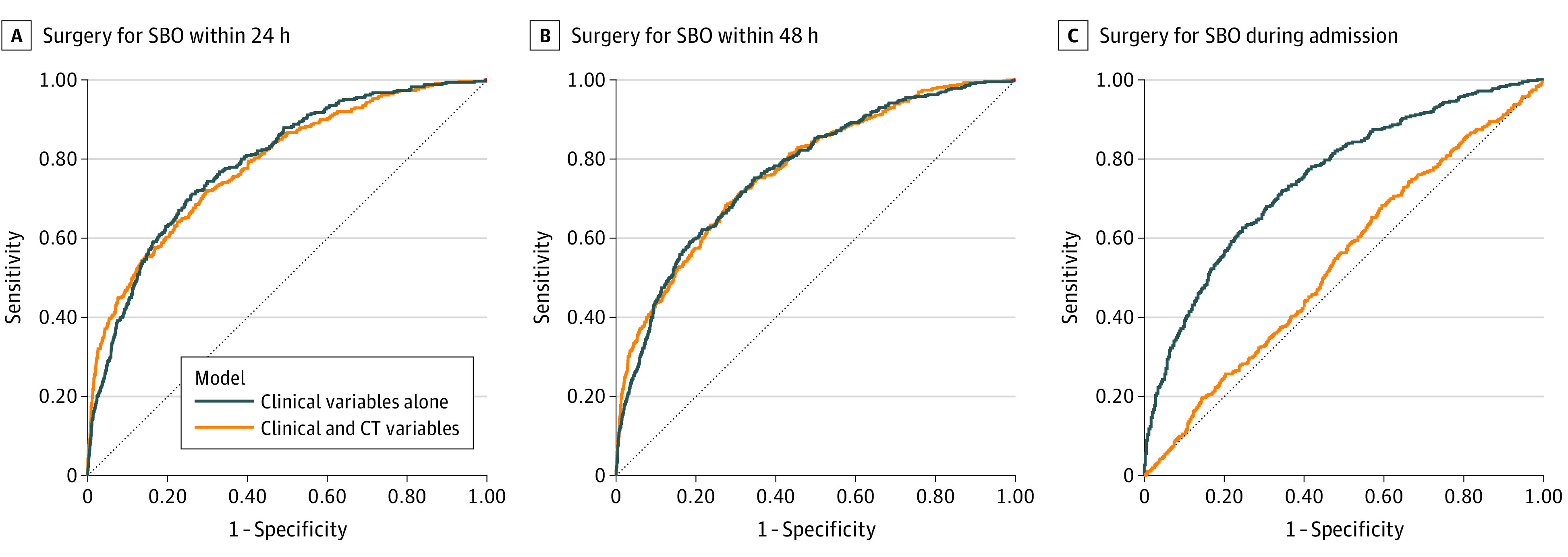
Estimated Need for Surgery Receiver operating characteristic curves are presented demonstrating the estimated need for operative treatment of patients with small bowel obstruction (SBO) A, within 24 hours of patient presentation; B, within 48 hours of patient presentation; and C, during admission. CT indicates computed tomography.

## Discussion

In this large multicenter comparative effectiveness research, a model combining clinical features and CT variables achieved equivalent performance in estimating the need for surgical SBO management within 24 or 48 hours of presentation as clinical variables alone. Furthermore, clinical features alone demonstrated greater accuracy in estimating the need for operative management at all times. These findings suggest that clinical features alone may be sufficient to identify the need for surgery in patients with SBO. Currently, more than 140 000 CT scans are performed on patients with suspected SBO annually.^5^ Our findings suggest that clinical features may risk-stratify and identify patients who require urgent surgical intervention. Alternative imaging modalities with lower cost and no radiation, such as point-of-care ultrasound (POCUS), have also demonstrated promising test characteristics in diagnosing SBO.^6^ A potential new workflow for assessing patients with suspected SBO may be to use POCUS for diagnostic imaging with clinical variables to determine the need for urgent surgery. The main limitation of our study is that it is not clear if all surgeries within our 24-hour cohort were urgent or emergent. Furthermore, classification or prospective studies are needed to determine CT’s role in determining operative intervention. Such studies may help elucidate a new workflow leading to lower health care costs and less radiation for this patient population.

## Notes

Supplement.
Data Sharing Statement
