# Supplementary material for: Clinical Features vs CT Findings to Estimate Need for Surgery in Small Bowel Obstruction
Source: JAMA Netw Open. 2023 Nov 2;6(11):e2341376. doi: 10.1001/jamanetworkopen.2023.41376 (PMC10623188; doi:10.1001/jamanetworkopen.2023.41376)
Supplement: Supplement. — Data Sharing Statement [file jamanetwopen-e2341376-s001.pdf]

Data Sharing Statement

Schulwolf. Clinical Features vs CT Findings to Estimate Need for Surgery in Small Bowel Obstruction. *JAMA Netw Open*. Published online November 3, 2023. doi:10.1001/ jamanetworkopen.2023.41376

.Data
Data available: No
